# Dimerized Hofstadter model in two-leg ladder quasi-crystals

**DOI:** 10.1038/s41598-024-59301-2

**Published:** 2024-04-16

**Authors:** Sara Aghtouman, Mir Vahid Hosseini

**Affiliations:** Department of Physics, Faculty of Science, University of Zanjan, Zanjan, 45371-38791 Iran

**Keywords:** Topological insulators, Quantum Hall

## Abstract

We theoretically study topological features, band structure, and localization properties of a dimerized two-leg ladder with an oscillating on-site potential. The periodicity of the on-site potential can take either rational or irrational values. We consider two types of dimerized configurations; symmetric and asymmetric models. For rational values of the periodicity as long as inversion symmetry is preserved both symmetric and asymmetric ladders can host topological phases. Additionally, the energy spectrum of the models exhibits a fractal structure known as the Hofstadter butterfly spectrum, dependent on the dimerization of the hopping and the strength of the on-site potential. In the case of irrational values for the periodicity, a metal-insulator phase transition occurs with small values of the critical strength of the on-site potential in the dimerized cases. Our models incorporate the effects of lattice configuration and quasi-periodicity, paving the way for establishing platforms that host both topological and non-topological phase transitions.

## Introduction

Non-trivial phase and quantum localization are the two most important features of topological states that have recently attracted much attention^1–3^. Such properties can serve as potential applications in quantum computing^4^. The quantum Hall effect^5,6^ harbors both of these features^7,8^. Topological edge states with energies within the gap are localized at the boundaries of the 2D quantum Hall lattice^9^. The topology of the edge states is linked to the bulk states and can be quantified by bulk topological invariants^10^. Also, thanks to the periodic motion of charge carriers in the presence of a gauge field within a periodic lattice structure of host materials, a fractal spectrum, known as Hofstadter’s butterfly spectrum, emerges^11,12^.

On the other hand, a 2D quantum Hall effect on the square lattice with the next-nearest-neighbor hopping^13–15^ can be reduced to a 1D quasi-periodic lattice^16,17^ exhibiting a metal-insulator transition. Subsequently, the topological edge states of 2D quantum Hall effect can be mapped into boundary states residing within certain gaps of a 1D quasi-periodic system^18^. Generally, in a 1D quasi-periodic lattice, in addition to the periodic discrete lattice, there is another periodic potential with different periodicity. Such quasi-periodic lattice systems have attracted much interest theoretically^19–22^ and experimentally^23–25^ due to providing a playground for studying band topology and localization in more realistic situations, namely, quasi-crystals^26^.

The quasi-periodic models have been further generalized to diverse systems^20,27–32^ revealing a mobility edge^33–35^ even beyond the tight-binding assumption^34,36^. Such systems have been used to demonstrate a self-duality symmetry^37,38^, an adiabatic pumping of boundary states^26,39^, quantum nonergodicity^40^, a many-body critical phase^41^, and localization effects^42–44,46^. The Hofstadter spectrum and topological phases have been proposed in a 1D quasi-periodic cold-atomic setting theoretically^18^ and observed experimentally in acoustic quasi-crystals^47^. The effect of periodically corrugations on a 1D lattice has been investigated resulting in the generation of topological states and butterfly spectrum^48^.

Furthermore, a non-trivial phase can be induced in 1D systems only by lattice dimerization^49,50^ being realized experimentally^51,52^. The dimerized 1D lattice has been generalized to a variety of configurations^53–57^ including, for example, spin-orbit coupling^58^ with characterized topological phase transitions^59^. The topological phase region has been extended due to invoking both spin-orbit coupling and Zeeman magnetic field^60,61^. Further, a non-zero Chern number has been obtained in the presence of both nearest-neighbor and next-nearest-neighbor hoppings^62^. Also, more sublattices per unitcell have been taken into account theoretically^63–65^ and experimentally^66^ which can host topological metal phase^67^. Also, it has been shown that coupled 1D dimerized lattices can host rich non-trivial topological features^68–72^ even with zero Berry curvature^73^. In the two-leg ladder geometry, the charge fractionalization has been characterized by Wilson lines^74^. Experimentally, topological bound states in a double dimerized chain based on split ring resonators^75^ and metamaterials^76^ have been observed.

Subsequently, the combination of the hopping dimerization and potential modulations can host topological states^77^, for instance, in Fibonacci quasi-crystals^78^ while there exists a topological equivalence between crystal and quasi-crystal band structures^78,79^. Also, in 2D lattices, it has been investigated the mutual effect of simultaneous modulations of hopping and on-site potential^80^ survives topological states in the localized regime^81^. For incommensurate hopping modulation case, Anderson-like localization has been found without mobility edge^39^. While for commensurate case, the emergence of topological gapless zero-energy modes has been reported^77^. In the two-leg dimerized lattice, the effect of including inter-leg coupling quasi periodicity and leaving on-site chemical potential modulations has been studied preserving particle-hole symmetry^82^.

However, a little attention has been paid to quasi-one-dimensional quasi-periodic systems^83^ with modulating hoppings and on-site potentials. In practice, particularly, for natural materials, it is easy to manipulate quasi-periodicity through on-site chemical potential via a spatially varying electric or back gate voltage. So, an alternative route is to implement quasi-periodic on-site chemical potentials preserving inversion symmetry. Subsequently, a topological quasi-periodic dimerized two-leg ladder would be realized.

In this paper, we study the topological features and localization character of a dimerized quasi 1D two-leg ladder with modulated on-site potential. The dimerization pattern can be either symmetric or asymmetric. The on-site potential has its own strength and frequency. We find that when the periodicity of the potential is such that it includes an even number of unitcells, the system has inversion symmetry. So the system hosts non-trivial topological edge states. The distribution of the topological regions in the phase diagram depends on either symmetric or asymmetric dimerization patterns. While if an odd number of unitcells lies in one period of the on-site potential, the system is topologically trivial regardless of dimerization pattern. We also show that the energy spectrum in terms of the on-site frequency has a fractal structure, which is known as the Hofstadter butterfly spectrum^11,16^. The spectrum displays a strong dependence not only on the type of dimerization pattern but also on the strength of the dimerization. For larger on-site potential strength, most of the states tend to be localized. For a irrational value of the frequency of the on-site potential, metal-insulator phase transition occurs at a certain strength of the on-site potential. Interestingly, in the presence of the dimerization, the critical value of the on-site potential strength decreases compared to the non-dimerized case.

The paper is organized as follows. In “Model and theory”, we introduce the model of the system having two different types; symmetric and asymmetric dimerized two-leg ladders. In “Symmetry analysis”, the symmetries of the system for commensurate and incommensurate cases are investigated. Accordingly, a relevant topological invariant is introduced in “Topological invariant”. In “Results and discussion”, the numerical results for rational and irrational values of the on-site potential frequency are presented. “Summary” includes the concluding remarks.

## Model and theory

**Figure 1:**
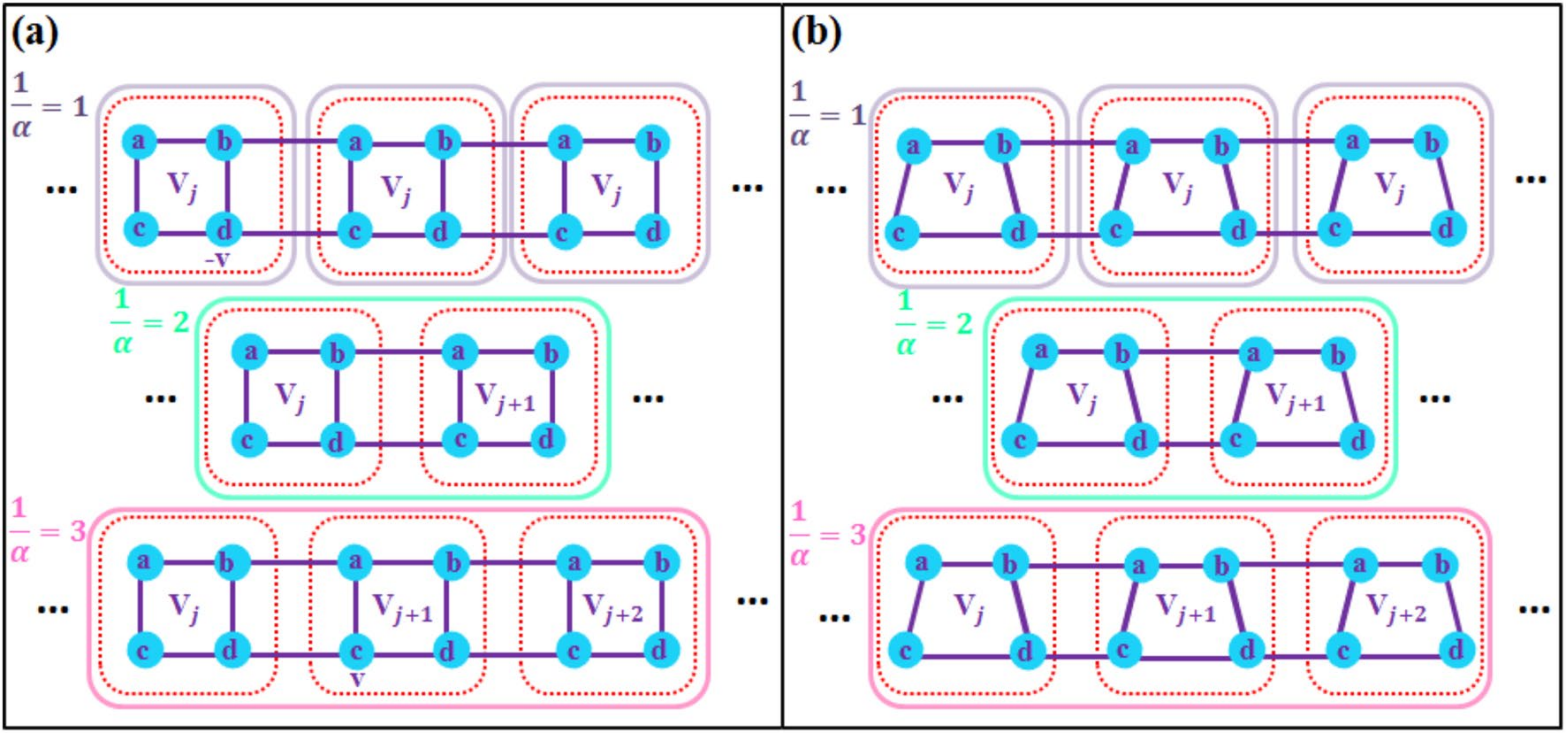
Schematic representation of a commensurate two-leg ladder containing four sublattices per unitcell, indicated by dashed boxes, with different periodicity ($1/\alpha =1,2,3$) for alternating on-site potentials, indicated by solid boxes. (**a**) Symmetric model with identical dimerized legs. (**b**) Asymmetric model with opposite dimerized legs.

We consider a dimerized two-leg ladder comprising of four sublattices per unitcell, as shown in Fig. 1, with an alternating on-site potential. The lattice structure can have two types of dimerization: (i) Symmetric dimerization where the upper and the lower legs have the same dimerization pattern (see Fig. 1a) and (ii) Asymmetric dimerization where the dimerization of the upper leg is opposite to that of the lower leg (see Fig. 1b). The tight-binding Hamiltonian describing the system is^44,68,69,82^

1 $$\begin{aligned} H=H_0+U, \end{aligned}$$

where the ladder Hamiltonian $H_0$ is

2 $$\begin{aligned} H_0&=\sum _{j=1}^{N}\biggr [t_1 (|a_{j}\rangle \langle b_{j}|+|b_{j}\rangle \langle a_{j}|) +t_2(|c_{j}\rangle \langle d_{j}|+|d_{j}\rangle \langle c_{j}|)+t_p(|a_{j}\rangle \langle c_{j}|+|c_{j}\rangle \langle a_{j}| +|b_{j}\rangle \langle d_{j}|+|d_{j}\rangle \langle b_{j}|) \biggr ]\nonumber \\&+\sum _{j=1}^{N-1}\biggr [t^{\prime }_{1}(|b_{j}\rangle \langle a_{j+1}|+|a_{j+1}\rangle \langle b_{j}|)+t^{\prime }_{2}(|d_{j}\rangle \langle c_{j+1}|+|c_{j+1}\rangle \langle d_{j}|)\biggr ], \end{aligned}$$

and the on-site potential *U* is

3 $$\begin{aligned} U=\sum _{j=1}^{N}V_{j}\biggr [|a_{j}\rangle \langle a_{j}|+|c_{j}\rangle \langle c_{j}|-|b_{j}\rangle \langle b_{j}|-|d_{j}\rangle \langle d_{j}|\biggr ], \end{aligned}$$

with

4 $$\begin{aligned} V_{j}=v \cos (2\pi \alpha j+\theta ). \end{aligned}$$

Here, $|x_{j}\rangle$ is the localized basis ket on the sublattice $x(=a, b, c, d)$ at the *j*th unitcell. $t_i$ and $t^{\prime }_i$ with $i=1,2$ are, respectively, the intra unitcell and the inter unitcell hopping amplitudes for the upper ($i=1$) and lower ($i=2$) legs. $t_p$ is the interleg hopping. *N* is the number of unitcells. $V_{j}$ is the on-site potential at the *j*th unitcell with *v* is the disorder strength, $\theta$ is a phase shift, and $1/\alpha$ is the periodicity of the potential. Note that $\alpha$ would take rational and irrational values for commensurate and incommensurate lattices, respectively. For the commensurate case, $1/\alpha$ determines how many unitcells lie within a one period of the on-site potential (see Fig. 1). While for the incommensurate case, an integer number of unitcells does not fit into the one period of the on-site potential.

For the symmetric model, all the intra unitcell and inter leg hoppings are equal to $t_{1,2}=t_p=t(1+\delta t)$ and the two inter unitcell hoppings are equal to $t^{\prime }_{1,2}=t(1-\delta t)$. But for the asymmetric form of the lattice dimerization, the intra unitcell hopping on the upper leg is equal to the inter unitcell hopping on the lower leg, $t_{1}=t^{\prime }_{2}=t(1-\delta t)$. Similarly, the other intra and inter unitcell hoppings are equal to each other as $t^{\prime }_{1}=t_{2}=t(1+\delta t)$ and the inter leg hoppings is $t_p=t(1+\delta t)$ with *t* being the magnitude of hopping. The dimerization strength is $\delta t=\delta _{0}\cos \vartheta$ with the amplitude $\delta _{0}$ and the phase $\vartheta$. We set *t* as the unit of the energy and $\delta _{0}=0.8$, without loss of generality.

If the eigenstate of the system, $|\Psi \rangle$, can be expanded by the localized basis $\psi _{x,j}$ as $|\Psi \rangle =\sum _j\bigoplus _{x=a,b,c,d}\psi _{x,j}|x_j\rangle$ so the eigenvalue equation $H|\Psi \rangle =E|\Psi \rangle$ can be written as

5 $$\begin{aligned} t_{1}\psi _{b,j}+t^{\prime }_{1}\psi _{b,j-1}+t_{p}\psi _{c,j}+V_{j}\psi _{a,j}=&E\psi _{a,j},\nonumber \\ t_{1}\psi _{a,j}+t^{\prime }_{1}\psi _{a,j+1}+t_{p}\psi _{d,j}-V_{j}\psi _{b,j}=&E\psi _{b,j},\nonumber \\ t_{p}\psi _{a,j}+t_{2}\psi _{d,j}+t^{\prime }_{2}\psi _{d,j-1}+V_{j}\psi _{c,j}=&E\psi _{c,j},\nonumber \\ t_{p}\psi _{b,j}+t_{2}\psi _{c,j}+t^{\prime }_{2}\psi _{c,j+1}-V_{j}\psi _{d,j}=&E\psi _{d,j}, \end{aligned}$$

where the $4N\times 4N$ Hamiltonian matrix is

6 $$\begin{aligned} \begin{aligned} H=&\begin{pmatrix}A_{1}&{}B&{}0&{}\dots &{}\dots &{}\dots &{}0\\ B^{\dagger }&{}A_{2}&{}B&{}0&{}\dots &{}\dots &{}0\\ 0&{}B^{\dagger }&{}A_{3}&{}B&{}\dots &{}\dots &{}0\\ \vdots &{}\ddots &{}\ddots &{}\ddots &{}\ddots &{}\ddots &{}\vdots \\ 0&{}\dots &{}0&{}B^{\dagger }&{}A_{N-2}&{}B&{}0\\ 0&{}\dots &{}\dots &{}0&{}B^{\dagger }&{}A_{N-1}&{}B\\ 0&{}\dots &{}\dots &{}\dots &{}0&{}B^{\dagger }&{}A_{N}\end{pmatrix}, \end{aligned} \end{aligned}$$

with

7 $$\begin{aligned} \begin{aligned} A_j=&\begin{pmatrix}V_{j}&{}t_{1}&{}t_{p}&{}0\\ t_{1}&{}-V_{j}&{}0&{}t_{p}\\ t_{p}&{}0&{}V_{j}&{}t_{2}\\ 0&{}t_{p}&{}t_{2}&{}-V_{j}\end{pmatrix}, B=&\begin{pmatrix}0&{}0&{}0&{}0\\ t^{\prime }_{1}&{}0&{}0&{}0\\ 0&{}0&{}0&{}0\\ 0&{}0&{}t^{\prime }_{2}&{}0 \end{pmatrix}. \end{aligned} \end{aligned}$$

## Symmetry analysis

In this section, our focus is on the investigation of the symmetry characteristics in both incommensurate and commensurate scenarios. As mentioned in the previous section the distinctive features between these two scenarios manifest in the value of $\alpha$. Additionally, it is crucial to note that in the incommensurate case, translational symmetry is violated, prompting an exploration of symmetry under open boundary conditions.

For the incommensurate case, e.g., $\alpha =(\sqrt{5}-1)/2$, Hamiltonian (6) for both symmetric and asymmetric models only exhibits time-reversal symmetry under open boundary condition, i.e., $\mathscr {T} H^* \mathscr {T}=H$, where the unitary part of time-reversal operator for the entire system is $\mathscr {T} =\sigma _{0_{4N}}$ and $\sigma _{0_i}$ is an $i\times i$ identity matrix. Furthermore, the value of $\alpha$ implies a lack of inversion symmetry, as the on-site potential’s periodicity does not align with an integer number of unitcells. Consequently, the absence of symmetry suggests that the system might not support topologically non-trivial phases.

For the commensurate case, there is a translational symmetry so Hamiltonian (Eq. 1) can be written in the Fourier space as

8 $$\begin{aligned} H=\sum _{k}\Psi _{k}^\dagger H(k)\Psi _{k}, \end{aligned}$$

where

9 $$\begin{aligned} \Psi _{k}^\dagger =(\psi _{a,1},\psi _{b,1},\psi _{c,1},\psi _{d,1},...,\psi _{a,\frac{1}{\alpha }},\psi _{b,\frac{1}{\alpha }},\psi _{c,\frac{1}{\alpha }},\psi _{d,\frac{1}{\alpha }})^\dagger , \end{aligned}$$

and the Hamiltonian matrix is

10 $$\begin{aligned} \begin{aligned} H(k)=&\begin{pmatrix}A_{1}&{}C&{}0&{}\dots &{}\dots &{}\dots &{}D\\ C^{\dagger }&{}A_{2}&{}C&{}0&{}\dots &{}\dots &{}0\\ 0&{}C^{\dagger }&{}A_{3}&{}C&{}\dots &{}\dots &{}0\\ \vdots &{}\ddots &{}\ddots &{}\ddots &{}\ddots &{}\ddots &{}\vdots \\ 0&{}\dots &{}0&{}C^{\dagger }&{}A_{\frac{1}{\alpha }-2}&{}C&{}0\\ 0&{}\dots &{}\dots &{}0&{}C^{\dagger }&{}A_{\frac{1}{\alpha }-1}&{}C\\ D^{\dagger }&{}\dots &{}\dots &{}\dots &{}0&{}C^{\dagger }&{}A_{\frac{1}{\alpha }}\end{pmatrix}, \end{aligned} \end{aligned}$$

with

11 $$\begin{aligned} \begin{aligned} C=Be^{ik}, \quad D=B^{\dagger }e^{-i\frac{k}{\alpha }}. \end{aligned} \end{aligned}$$

The Bloch condition in the momentum space, i.e., $\psi _{x,m+\frac{1}{\alpha }}=e^{i\frac{k}{\alpha }}\psi _{x,m}$, is used. In the following, we will investigate symmetries of the system with either even or odd numbers of unitcells, e.g., $1/\alpha =2$ and $1/\alpha =3$, for both symmetric and asymmetric cases.

### Symmetric ladder

For the symmetric ladder (see Fig.1a), $t_1=t_2=t_p$, $t^{\prime }_1=t^{\prime }_2$, we take $1/\alpha =2$ implying that $V_1=-V_2$. So Hamiltonian (10) can be re-written in the basis $\Psi _{k}^\dagger =(\psi _{a,1},\psi _{b,1},\psi _{c,1},\psi _{d,1},\psi _{a,2},\psi _{b,2},\psi _{c,2},\psi _{d,2})^\dagger$ as

12 $$\begin{aligned} H(k)= & {} \begin{pmatrix} A_1 &{} C+D \\ C^{\dagger }+D^{\dagger } &{} A_2 \end{pmatrix}, \end{aligned}$$

whose elements are defined in Eqs. (7) and (11). It is easy to show that Hamiltonian (Eq. 12) exhibits time-reversal and inversion symmetry. The time-reversal symmetry, i.e., $\mathscr {T}_{i} H^*(k) \mathscr {T}_{i}=H(-k)$, has the corresponding unitary operators $\mathscr {T}_{1} =\sigma _{0_{2}}\otimes (\sigma _{x}\otimes \sigma _{0_{2}})$ and $\mathscr {T}_{2}=\sigma _{0_8}$. In addition, the inversion symmetry, i.e., $\Pi _i H(k) \Pi _i=H(-k)$, is characterized by two distinctive operators $\Pi _{1}=\sigma _{x}\otimes (\sigma _{x}\otimes \sigma _{x})$ and $\Pi _{2}=\sigma _{x}\otimes (\sigma _{0_2}\otimes \sigma _{x})$. Here $\sigma _{x}$ represents the *x* component of the Pauli matrices.

The presence of the two inversion operators suggests the existence of an additional symmetry, namely, the exchange symmetry^72^. The exchange operator can be expressed as the product of the two operators of inversion symmetry, i.e., $Y = \Pi _{1}\cdot \Pi _{2}=\sigma _{0_2}\otimes (\sigma _{x}\otimes \sigma _{0_2})$. This operator exchanges the two legs of the ladder and their corresponding sublattices as

13 $$\begin{aligned} Y\Psi \rightarrow \Psi ^{\prime }=&\begin{pmatrix}&{}\psi _{c,1}&{}\\ {} &{}\psi _{d,1}&{}\\ {} &{}\psi _{a,1}&{}\\ {} &{}\psi _{b,1}&{}\\ {} &{}\psi _{c,2}&{}\\ {} &{}\psi _{d,2}&{}\\ {} &{}\psi _{a,2}&{}\\ {} &{}\psi _{b,2}&{}\end{pmatrix}. \end{aligned}$$

Obviously, Hamiltonian (12) can commute with the exchange operator, $\left[ Y,H(k)\right] =0$ and it can be brought into the block diagonal form as

14 $$\begin{aligned} \tilde{H}=XHX^{-1}= & {} \begin{pmatrix} h_{-}&{} 0 \\ 0 &{} h_{+} \end{pmatrix}, \end{aligned}$$

where

15 $$\begin{aligned} h_{\pm }=&\begin{pmatrix}\pm t_{1}+V_{1}&{}t_{1}&{}0&{}t^{\prime }_{1}e^{2ik}\\ t_{1}&{}\pm t_{1}-V_{1}&{}t^{\prime }_{1}e^{-ik}&{}0\\ 0&{}t^{\prime }_{1}e^{ik}&{}\pm t_{1}-V_{1}&{}t_{1}\\ t^{\prime }_{1}e^{-i2k}&{}0&{}t_{1}&{}\pm t_{1}+V_{1}\end{pmatrix}, \end{aligned}$$

and

16 $$\begin{aligned} X=&\begin{pmatrix}0&{}0&{}0&{}0&{}0&{}-1&{}0&{}1\\ 0&{}0&{}0&{}0&{}-1&{}0&{}1&{}0\\ 0&{}-1&{}0&{}1&{}0&{}0&{}0&{}0\\ -1&{}0&{}1&{}0&{}0&{}0&{}0&{}0\\ 0&{}0&{}0&{}0&{}0&{}1&{}0&{}1\\ 0&{}0&{}0&{}0&{}1&{}0&{}1&{}0\\ 0&{}1&{}0&{}1&{}0&{}0&{}0&{}0\\ 1&{}0&{}1&{}0&{}0&{}0&{}0&{}0\end{pmatrix}. \end{aligned}$$

This means that in the presence of such symmetry, one can decompose the system into two decoupled subsystems. Therefore, topological properties of each subsystem is independent of the other one. It is worthwhile noting that each subsystem (Eq. 15) has the inversion symmetry, i.e., $\Pi ^{\prime } h(k) \Pi ^{\prime }=h(-k)$, with $\Pi ^{\prime }=\sigma _{x}\otimes \sigma _{x}$ being the subsystem inversion operator.

In general, for any even number of $1/\alpha = M$ ($M=2n$, $n \in \mathbb {N}$) under the condition $\theta =(\frac{1}{2\alpha }-1)\pi \alpha$, the system exhibits inversion symmetry. However, in the case of $1/\alpha =2$, inversion symmetry exists for all values of $\theta$ without any additional conditions. This arises from the reduction of Eq. (4) to $V_{j}=(-1)^jv \cos \theta$. The inversion symmetry operators for the entire system take the forms $\Pi _{1}=\sigma _{x_{4M}}$ and $\Pi _{2}=\sigma _{x}\otimes (\sigma _{0_{M}}\otimes \sigma _{x})$ where17While for the subsystems, the inversion operator is $\Pi ^{\prime }=\sigma _{x_{2M}}$. Besides, for the entire system the time-reversal operators take the forms $\mathscr {T}_{1} =\sigma _{0_2}\otimes (\sigma _{x}\otimes \sigma _{0_{M}})$ and $\mathscr {T}_{2}=\sigma _{0_{4M}}$.

Now let’s check the case $1/\alpha =3$ with the Hamiltonian that is re-written as

18 $$\begin{aligned} H(k)= & {} \begin{pmatrix} A_{1}&{}C&{}D\\ C^{\dagger }&{}A_{2}&{}C\\ D^{\dagger }&{}C^{\dagger }&{}A_{3} \\ \end{pmatrix}, \end{aligned}$$

whose elements are defined in Eqs. (7) and (11). The basis is $\Psi _{k}^\dagger =(\psi _{a,1},\psi _{b,1},\psi _{c,1},\psi _{d,1},...,\psi _{a,3},\psi _{b,3},\psi _{c,3},\psi _{d,3})^\dagger$. In this case, although there is no inversion symmetry resulting in non-topological subsystems, one still can find the exchange operator $Y =\sigma _{0_3}\otimes (\sigma _{x}\otimes \sigma _{0_2})$ exchanging the two legs of the ladder and their corresponding sublattices as $$Y\Psi \rightarrow |\Psi ^{\prime }\rangle =\bigoplus _{\begin{array}{c} \text {x=c,d,a,b}\\ \text {y=1,2,3} \end{array}}\psi _{x,y}|x\rangle \otimes |y\rangle$$. Similarly, in the basis of the exchange operator, i.e., *X*, the Hamiltonian (Eq. 18) can be block-diagonalized as

19 $$\begin{aligned} \tilde{H}=XHX^{-1}= & {} \begin{pmatrix} h_{-}&{} 0 \\ 0 &{} h_{+} \end{pmatrix}, \end{aligned}$$

where

20 $$\begin{aligned} \begin{aligned} h_{\pm }=\begin{pmatrix}\pm t_{1}-V_{3}&{}t_{1}&{}0&{}0&{}0&{}t^{\prime }_{1}e^{3ik}\\ t_{1}&{}\pm t_{1}+V_{3}&{}t^{\prime }_{1}e^{-ik}&{}0&{}0&{}0\\ 0&{}t^{\prime }_{1}e^{ik}&{}\pm t_{1}-V_{2}&{}t_{1}&{}0&{}0\\ 0&{}0&{}t_{1}&{}\pm t_{1}+V_{2}&{}t^{\prime }_{1}e^{-ik}&{}0\\ 0&{}0&{}0&{}t^{\prime }_{1}e^{ik}&{}\pm t_{1}+V_{1}&{}t_{1}\\ t^{\prime }_{1}e^{-3ik}&{}0&{}0&{}0&{}t_{1}&{}\pm t_{1}-V_{1}\end{pmatrix}, \end{aligned} \end{aligned}$$

and

21 $$\begin{aligned} X=\begin{pmatrix} \begin{array}{rrrrrrrrrrrr} \!\!\!0&{}0&{}0&{}0&{}0&{}0&{}0&{}0&{}0&{}-1&{}0&{}1\\ \!\!\!0&{}0&{}0&{}0&{}0&{}0&{}0&{}0&{}-1&{}0&{}1&{}0\\ \!\!\!0&{}0&{}0&{}0&{}0&{}-1&{}0&{}1&{}0&{}0&{}0&{}0\\ \!\!\!0&{}0&{}0&{}0&{}-1&{}0&{}1&{}0&{}0&{}0&{}0&{}0\\ \!\!\!0&{}-1&{}0&{}1&{}0&{}0&{}0&{}0&{}0&{}0&{}0&{}0\\ \!\!\!-1&{}0&{}1&{}0&{}0&{}0&{}0&{}0&{}0&{}0&{}0&{}0\\ \!\!\!0&{}0&{}0&{}0&{}0&{}0&{}0&{}0&{}0&{}1&{}0&{}1\\ \!\!\!0&{}0&{}0&{}0&{}0&{}0&{}0&{}0&{}1&{}0&{}1&{}0\\ \!\!\!0&{}0&{}0&{}0&{}0&{}1&{}0&{}1&{}0&{}0&{}0&{}0\\ \!\!\!0&{}0&{}0&{}0&{}1&{}0&{}1&{}0&{}0&{}0&{}0&{}0\\ \!\!\!0&{}1&{}0&{}1&{}0&{}0&{}0&{}0&{}0&{}0&{}0&{}0\\ \!\!\!1&{}0&{}1&{}0&{}0&{}0&{}0&{}0&{}0&{}0&{}0&{}0 \end{array} \end{pmatrix}. \end{aligned}$$

For Hamiltonian (Eq. 18) the time-reversal operators take the forms $\mathscr {T}_{1} =\sigma _{0_3}\otimes (\sigma _{x}\otimes \sigma _{0_{2}})$ and $\mathscr {T}_{2}=\sigma _{0_{12}}$. In general, for any odd number of $1/{\alpha }=M$ ($M=2n+1$) there is no inversion symmetry. Moreover, the time-reversal symmetry operators are $\mathscr {T}_{1} =\sigma _{0_{M}}\otimes (\sigma _{x}\otimes \sigma _{0_{2}})$ and $\mathscr {T}_{2}=\sigma _{0_{4M}}$.

### Asymmetric ladder

For asymmetric model, with $t_1=t^{\prime }_2, t_2=t^{\prime }_1$ and $1/{\alpha }=2$, we get the general form of Hamiltonian (Eq. 12). In this case, the only inversion symmetry operator is $\Pi =\sigma _{x}\otimes (\sigma _{0_2}\otimes \sigma _{x})$. So, there is no exchange symmetry. Subsequently, the system cannot be decomposed into subsystems. Also, the time-reversal symmetry operator is $\mathscr {T} =\sigma _{0_8}$. So, for the entire system with $1/{\alpha }=M$ ($M=2n$) the inversion and time-reversal symmetry take the forms $\Pi =\sigma _{x}\otimes (\sigma _{0_{M}}\otimes \sigma _{x})$ and $\mathscr {T} =\sigma _{0_{4M}}$, respectively. Also at $1/{\alpha }=3$, with applying the conditions of the asymmetric ladder, we get Hamiltonian (Eq. 18) supporting only time-reversal symmetry with operator $\mathscr {T} =\sigma _{0_{12}}$. In general, for any odd number of $1/{\alpha }=M$ ($M=2n+1$) there is not any inversion symmetry and the time-reversal operator takes the general form $\mathscr {T} =\sigma _{0_{4M}}$.

## Topological invariant

As already discussed above, in the presence of the on-site potential with a period maintaining an even number of unitcells, the chain has inversion symmetry and, as will be shown below, it would hosts non-trivial topological phases^84^. So we calculate the *Z* invariant^85,86^, as a relevant invariant for the ladder, defined by

22 $$\begin{aligned} Z:= \sum _{j} \sum _{i} |\varepsilon _{1ij}-\varepsilon _{2ij}|, \end{aligned}$$

where $\varepsilon _{1ij}$ and $\varepsilon _{2ij}$ are the number of negative parities of the band structure at the super symmetry points $k=0$ and $k=\pi$ in the *i*th band gap of the *j*th subspace, respectively.

Furthermore, in order to investigate the localization property of the states, we calculate the inverse participation ratio (IPR) of each state as^87^

23 $$\begin{aligned} IPR=\frac{\sum _{j}\sum _{x}|\psi _{x,j}|^{4}}{(\sum _{j}\sum _{x}|\psi _{x,j}|^{2})^{2}}, \end{aligned}$$

where $\psi _{x,j}$ is defined above. When IPR tends to 0, the states are extended and for IPR values close to 1, the states are localized. We will also calculate the mean IPR (MIPR)^44^ associated with the ground state over 10 phases shift randomly in order to reveal metal-insulator phase transition.

**Figure 2:**
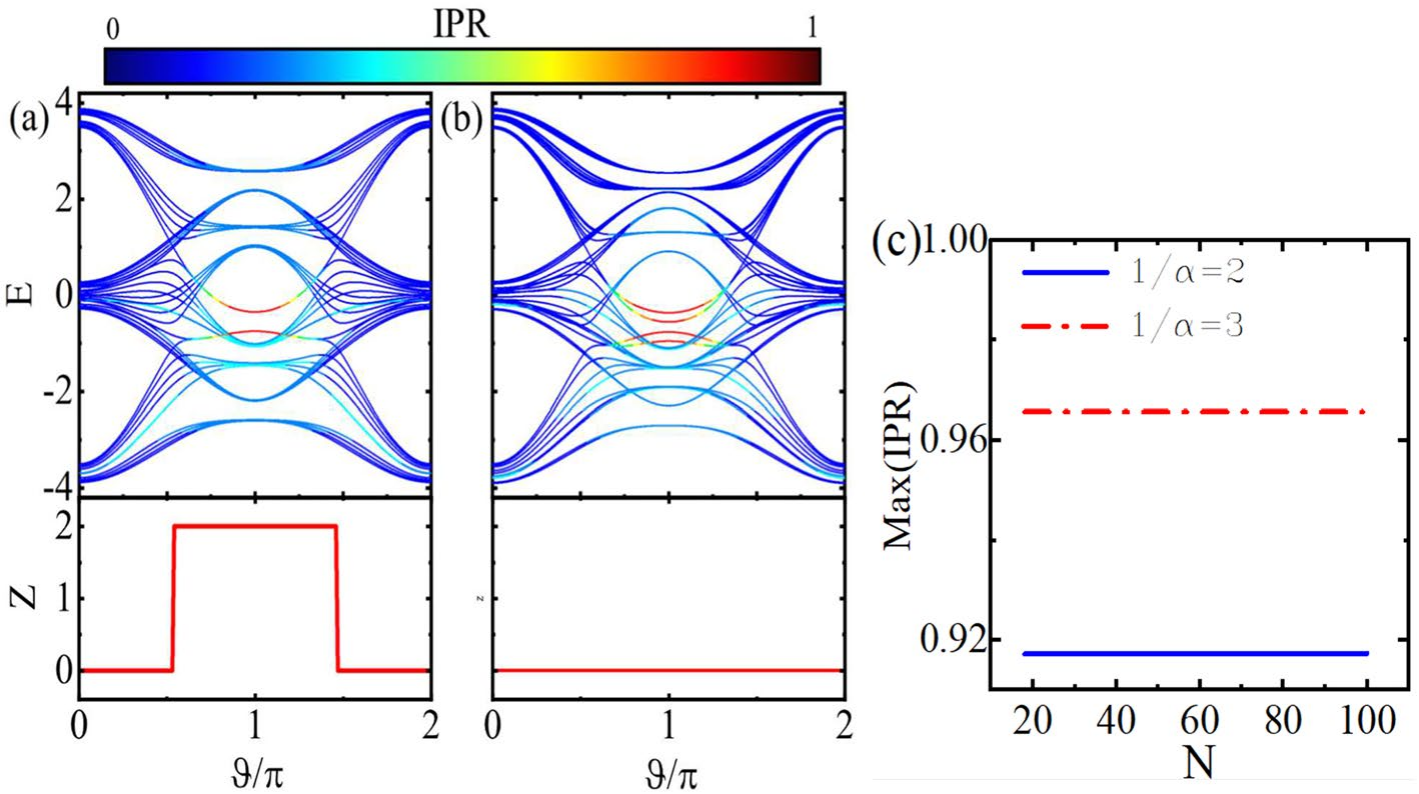
(Color online) Energy spectrum and the topological invariant *Z* of the symmetric ladder versus $\vartheta$ for (**a**) $1/\alpha =2$ and (**b**) $1/\alpha =3$. (**c**) Maximum value of IPR as a function of the unitcell number N for different values of $1/\alpha$ with $\vartheta =\pi$. Here, $\theta =\pi /4$ and $v=0.8$.

## Results and discussion

In our model, we first present the results of the commensurate case for rational values of $\alpha$, revealing the non-trivial topological properties of bulk systems. Then, we discuss the effect of dimerization on the metal-insulator transition point^17^ for the incommensurate case at $\alpha =(\sqrt{5}-1)/2$. In the following, we investigate the numerically calculated results for both symmetric and asymmetric models in detail.

### Rational value of $\alpha$

In Fig. 2a, b, the energy spectra and the relevant topological invariant of the symmetric model are shown as a function of $\vartheta$ for $\theta =\pi /4$ and $v=0.8$. It can be seen from Fig. 2a, with the value of $1/\alpha =2$, as $\vartheta$ varies, topological phase transitions can occur at $\vartheta =\pi /2,3\pi /2$. Subsequently, the *Z* invariant shows a non-trivial value between $\vartheta =\pi /2$ and $\vartheta =3\pi /2$, resulting in the appearance of doubly degenerate localized edge states not only in the gap but also inside the topological bulk states. Also, the Z invariant gets the value 2. Because, as already discussed, the system can be decomposed into two subsystems, each with its own set of topological edge states positioned either in the bulk or gap of the other subsystem. Such edge states are finite-energy ones being protected by the inversion symmetry of the subsystem Hamiltonian. But by changing the periodicity of the on-site potential covering an odd number of unitcells, for instance, $1/\alpha =3$, the inversion symmetry will be broken. Subsequently, as it is shown in Fig. 2b, in this case, the edge states are no longer protected topologically and the topological invariant takes trivial values for all values of the dimerization $\vartheta$. In Fig. 2c, the IPR of the most localized states is depicted as a function of unitcell number *N* for both even and odd numbers of $1/\alpha$. In either case, it is evident that the localized states remain unaffected as the system size increases. This implies that the induced localization is scale-free^45^.

**Figure 3:**
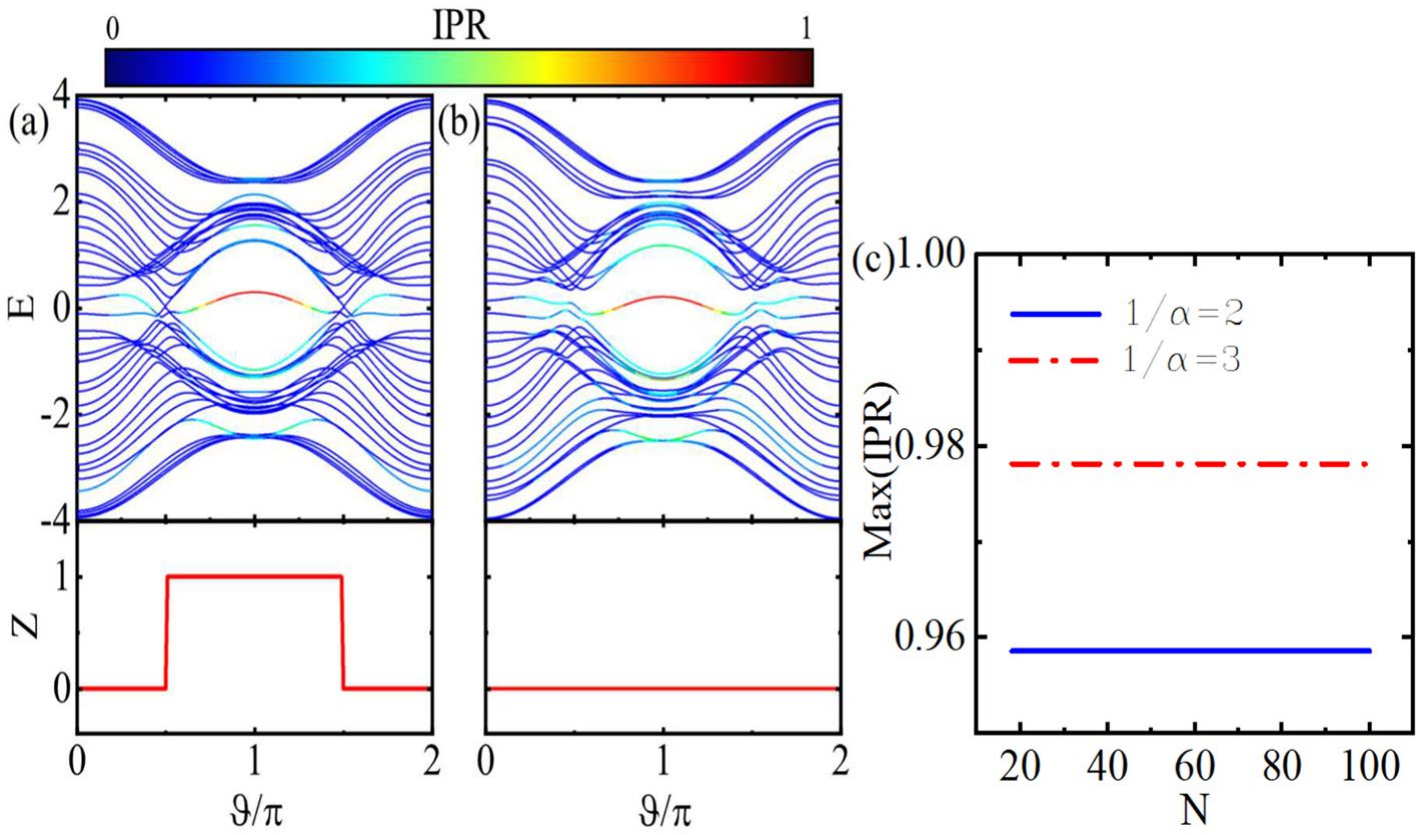
(Color online) Energy spectrum and topological invariant *Z* of the asymmetric ladder versus $\vartheta$ for (**a**) $1/\alpha =2$ and (**b**) $1/\alpha =3$. (**c**) Maximum value of IPR as a function of the unitcell number N for different values of $1/\alpha$ with $\vartheta =\pi$. Here, $\theta =\pi /4$ and $v=0.8$.

**Figure 4:**
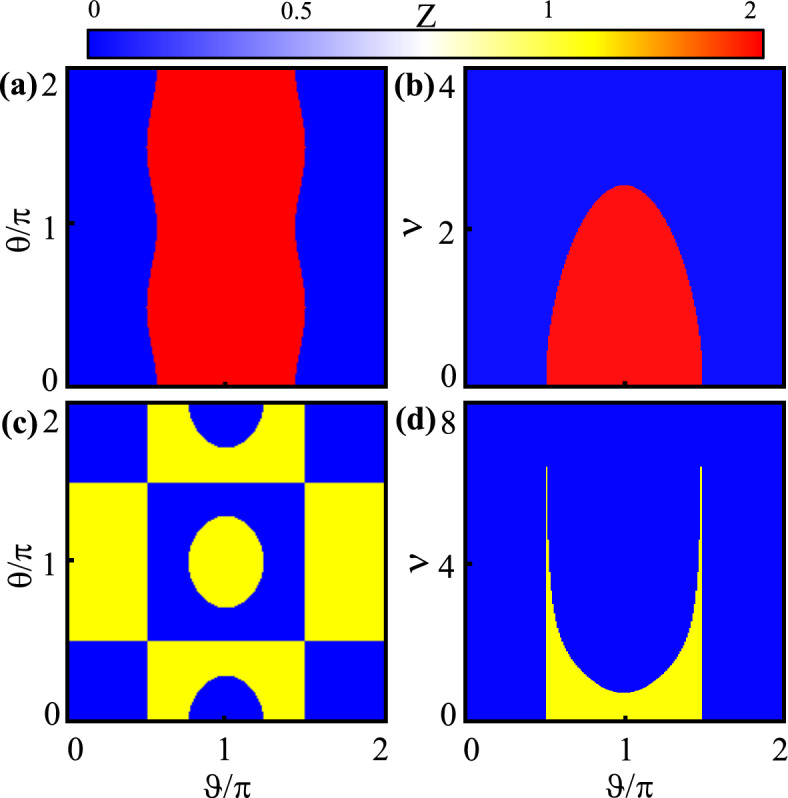
(Color online) Topological phase diagrams for symmetric (top row) and asymmetric (bottom row) ladders as functions of ($\theta$, $\vartheta$) (left column) with $v=0.8$, and of (*v*, $\vartheta$) (right column) with $\theta =\pi /4$. Here, $1/\alpha =2$.

Figure 3a, b show the dependence of the band structure and the topological *Z* invariant of the asymmetric model on $\vartheta$. As shown in Fig. 3a, for $1/\alpha =2$, with the opening of the band gap, doubly degenerate edge states appear in the gap and the *Z* number gets a non-zero value due to the presence of the inversion symmetry of the whole system. In this case, since the system cannot be decomposed into subsystems, unlike the symmetric case, the Z invariant takes the value 1. For $1/\alpha =3$, as depicted in Fig. 3b, the energy spectrum is topologically trivial. Because, the system lacks the inversion symmetry. Furthermore, the bulk gap closing does not occur around $\vartheta =\pi /2,3\pi /2$ which is in contrast to the symmetric ladder case. Figure 3c shows the maximum value of the IPR of the states versus the system length. Similar to the symmetric case, in the present case, the observed localization remains independent of the system size, irrespective of whether $1/\alpha$ is even or odd.

**Figure 5:**
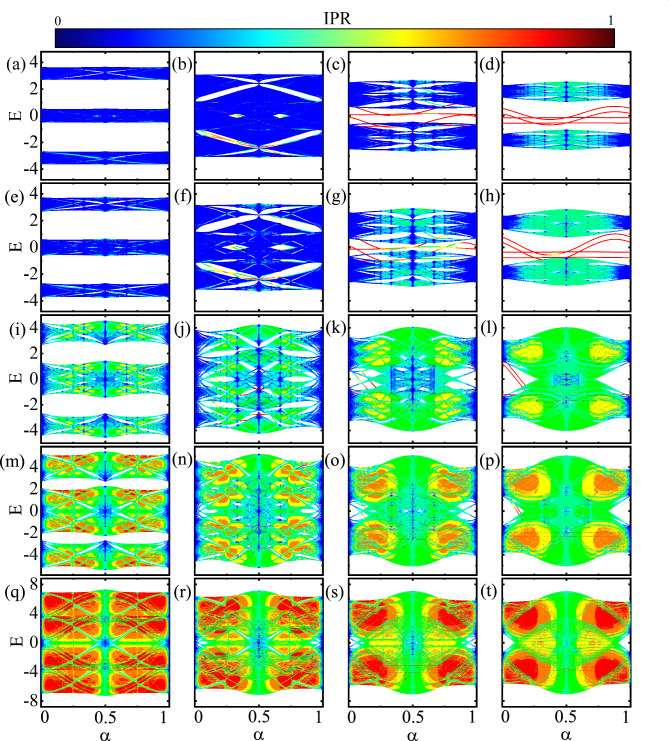
(Color online) Energy spectrum of symmetric model versus $\alpha$ for $v=0.5$ (**a–d**), $v=0.8$ (**e–h**), $v=2$ (**i–l**), $v=3$ (**m–p**), and $v=5$ (**q–t**). Also, $\vartheta =\pi /4,\pi /2, 3\pi /4, \pi$ for the first, the second, the third, and the forth columns, respectively. Here, $\theta =\pi /4$.

The topological phase diagram of the symmetric (asymmetric) ladder is displayed in the top (bottom) row of Fig. 4 for $1/\alpha =2$. In the symmetric ladder, the topological and the trivial regions are shown in red and blue colors, respectively. In Fig. 4a, the Z invariant is shown as functions of $\theta$ and $\vartheta$ with $v=0.8$. The middle area around $\pi /2<\vartheta <3\pi /2$ for any value of $\theta$ is covered by a topological region. In Fig. 4b, the Z invariant is depicted as functions of *v* and $\vartheta$ with $\theta =\pi /4$. Again around $\pi /2<\vartheta <3\pi /2$, there is a topological region but below the value $v=2.5$.

**Figure 6:**
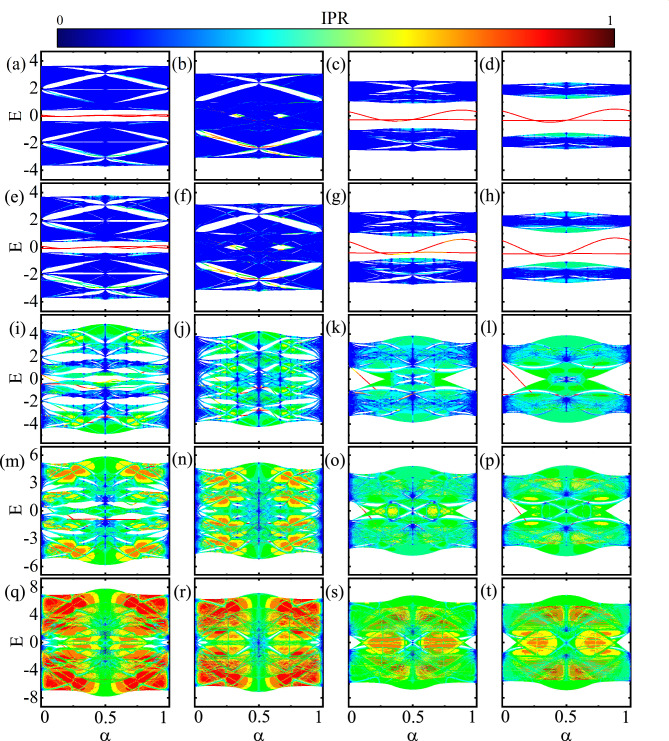
(Color online) Energy spectrum of asymmetric model versus $\alpha$ for $v=0.5$ (**a–d**), $v=0.7$ (**e–h**), $v=2$ (**i–l**), $v=3$ (**m–p**) and $v=5$ (**q–t**). Also, $\vartheta =\pi /4,\pi /2, 3\pi /4, \pi$ for the first, the second, the third, and the forth columns, respectively. Here, $\theta =\pi /4$.

Similar to Fig. 4a, b, the topological phase diagram of the asymmetric ladder with $1/\alpha =2$ is illustrated in Fig. 4c, d. The figures show the non-trivial and trivial regions in yellow and blue colors, respectively. As can be seen in Fig. 4c, topologically non-trivial regions can be found not only in the central region around $\theta \approx \vartheta \approx \pi$ but also in the peripheral region. In Fig. 4d, we can see that the non-trivial region around $\pi /2<\vartheta <3\pi /2$ is extended up to the value *v*=7. However, in contrast to Fig. 4b, the topological region around $\vartheta \approx \pi$ has the lowest value of *v*.

The commensurate scenario can be extended by taking into account the rational values as $\alpha = \frac{p}{q}$, where *p* and *q* are integers and coprime. By incorporating the periodic potential $V_{j}$ with a period *q* under open boundary conditions, we can solve the eigenvalue problem in relation to $\alpha$ using Eq. (6) with $N=q=199$. Subsequently, through the solution of the system and the computation of $\text {IPR}$, we obtain the fractal spectrum, known as Hofstadter spectra, including edge states. The Hofstadter spectra of the symmetric and asymmetric ladders at $\theta =\pi /4$ are illustrated in Figs. 5 and 6, respectively.

Figure 5 shows the energy spectra of the symmetric model as a function of $\alpha$. From the top row to the bottom row, the disorder strength is $v=0.5, 0.8, 2, 3, 5$ and from the left column to the right column the dimerization is $\vartheta =\pi /4, \pi /2, 3\pi /4, \pi$. For $v=0.5$ and $v=0.8$, as shown on the first two rows of Fig. 5, at $\vartheta =\pi /4$, the Fermi energy is bulk gapless and there are two main gaps. At $\vartheta =\pi$, the band widths increase and the main gaps reduces to partially narrow gaps. As $\vartheta$ increases the band widths decrease again such that a substantial main bulk gap appears around the Fermi energy. For the larger values of the disorder strength $v=2$ and $v=3$ (the third and the forth rows), at $\vartheta =\pi /4$, the two main gaps are decreased. Also, for larger values of $\vartheta$ the bands join together providing a bulk gapless system. For $v=5$ (the fifth row), the spectrum is almost always gapless regardlss of the value of $\vartheta$.

Figure 6 shows the Hofstadter butterfly spectrum of the asymmetric model versus $\alpha$ for different values of *v* and $\vartheta$. The panels from the left column to the right column have the values $\vartheta =\pi /4,\pi /2,3\pi /4,\pi$, and from the top row to the bottom row have the values $v=0.5, 0.7, 2,3, 5$. From the first two rows, one can see that, unlike the symmetric ladder case, for $\vartheta =\pi /4$, there a main gap at the Fermi level $E=0$. But, similar to the symmetric case, for $\vartheta =\pi /2$ the system becomes gapless at the Fermi level. Because, at $\vartheta =\pi /2$, the system is non-dimerized so there is no difference between the two models. For $\vartheta =3\pi /4,\pi$ a considerable gap opens at the Fermi level, and at the same time, the band widths decease. Moreover, at large disorder potential strength, $v=2,3,5$, the width of the band becomes wider so that the valence and conduction bands merge together and the band gap closes.

As evident from both Figs. 5 and 6, when the values of *v* are below certain thresholds, delocalized states dominate, while for larger values of *v*, localized states become more prominent. This observation suggests the existence of a transition point, which will be investigated further below. Moreover, typically, for $\alpha =0, 1/2, 1$, the delocalized states can persist even at large values of *v*.

### Irrational value of $\alpha$

**Figure 7:**
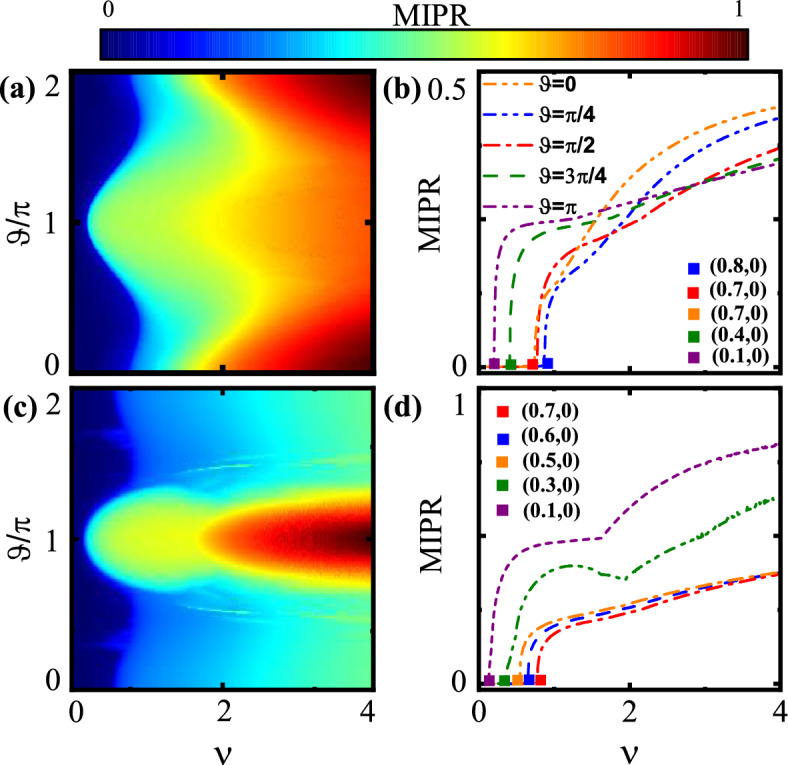
(Color online) Metal-insulator phase diagram of (**a**) the symmetric and (**c**) the asymmetric models as functions of dimerization parameter $\vartheta$ and the disorder strength *v*. MIPR of (**b**) the symmetric and (**d**) the asymmetric ladders versus disorder strength *v* for different values of $\vartheta$.

We now consider the model under open boundary conditions and solve Eq. (6) for the incommensurate case with irrational value of $\alpha =(\sqrt{5}-1)/2$. We numerically evaluate the MIPR related to the ground states. The density plot of the phase diagram as functions of *v* and $\vartheta$, for symmetric ladder is shown in Fig. 7a. The figure shows the delocalized states with blue color and the localized states with red color. Interestingly, the critical value of *v*, at which metal to insulator phase transition occurs, strongly depends on the dimerization strength. For $\vartheta \approx \pi$, i.e., the intra unitcell hoppings $t_{1,2}$ are smaller that the inter unitcell hoppings $t^{\prime }_{1,2}$, the critical value *v* reaches to its smallest value. This implies that in this case only small values of disorder potential can make the system an insulator. Furthermore, for $\vartheta \approx 0,2\pi$ and large enough *v*, the states are the most localized ones. Figure 7b is the cross-section of the panel (a) and shows the dependence of MIPR on *v* for specific values of $\vartheta$. It is clear that as $\vartheta$ decreases from $\pi$ to 0 the value of the transition point increases and then slightly decreases.

For asymmetric ladder, the density plot of the phase diagram as functions of *v* and $\vartheta$, is shown in Fig. 7c. The dark blue region indicates metallic states. Similar to the symmetric ladder case, there is a non-monotonic behavior of the critical value of *v* versus dimerization such that the lowest critical value of *v* is around $\vartheta \approx \pi$. In contrast, for large enough disorder strength, the most localized states are around $\vartheta \approx \pi$. To clarify the phase diagram, we have plotted the MIPR as a function of the *v* with various values of $\vartheta$ as shown in Fig. 7d. Similarly, in this ladder, with raising $\vartheta$, from 0 to $\pi$ the transition point gets larger values, but for $\vartheta >\pi /2$, the increasing of the $\vartheta$ makes the transition point to get smaller values with the smallest one at $\vartheta =\pi$.

From Fig. 7, for both ladders, as a result, one finds that the overall critical value of the disorder strength is smaller than that of the original Aubry-Andre model, i.e., 1D non-dimerized chain with one sublattice per unitcell^17^. This can be attributed to the existence of more sublattices per unitcell in our model compared to the Aubry-Andre chain. Moreover, in overall, a small dimerization parameter, denoted as $\delta t$, results in a lower value for the transition point. Consequently, dimerization renders the metallic states more unstable than in the non-dimerized case, favoring the formation of the insulating phase.

## Summary

We conducted a study on the topological and localization properties of the two-leg ladder with symmetric and asymmetric dimerization configurations, incorporating on-site energies. The on-site potential exhibits an oscillatory behavior along the chain. The lattice can be either commensurate or incommensurate, depending on whether the frequency of the on-site potential takes rational or irrational values. In the former case, an integer number of unitcells can fit within one period of the potential, while in the latter case, one period of the on-site potential does not cover an integer number of unitcells.

We calculated the band structure and phase diagrams for both symmetric and asymmetric models. Our findings indicate that both models can host topologically non-trivial phases in the commensurate case when there is an even number of unitcells in one period of the on-site potential. Under such conditions, inversion symmetry can be established, protecting the symmetry-protected topological phases. Additionally, we obtained the fractal spectrum, known as Hofstadter’s butterfly, for the symmetric and asymmetric models with different dimerization and on-site potential strengths. Our analysis revealed that the states of the fractal spectrum tend to be more delocalized at on-site potential strengths less than certain values, while becoming more localized for sufficiently large on-site potential strengths.

Subsequently, we investigated incommensurate lattices, identifying metal-insulator transition points influenced by the dimerization strength. The critical value of on-site potential strength for the transition point in the non-dimerized case is larger than that in the dimerized case, and vice versa.

## Data Availability

All data generated or analyzed during this study are included in this published article.

## References

[1] Hasan MZ, Kane CL (2010). Topological insulators. Rev. Mod. Phys..

[2] Qi X-L, Zhang S-C (2011). Topological insulators and superconductors. Rev. Mod. Phys..

[3] Sato M, Ando Y (2017). Topological superconductors. Rep. Prog. Phys..

[4] Nayak C, Simon SH, Stern A, Freedman M, Sarma S. Das (2008). Non-abelian anyons and topological quantum computation. Rev. Mod. Phys..

[5] Hansson, T.H., Hermanns, M., Simon, S.H. & Viefers, S.F. Quantum hall physics-hierarchies and CFT techniques. *Rev. Mod. Phys.* **89**, 025005 (2017).

[6] von Klitzing K (2017). Quantum Hall effect. Annu. Rev. Condens. Matter Phys..

[7] Thouless DJ, Kohmoto M, Nightingale MP, den Nijs M (1982). Quantized Hall conductance in a two-dimensional periodic. Phys. Rev. Lett..

[8] Faist J (1994). Quasicrystals.

[9] Kraus YE, Zilberberg O (2018). High-order topological insulators from high-dimensional Chern insulators. Nat. Phys..

[10] Zilberberg O (2021). Opt. Mater. Exp..

[11] Hofstadter DR (1976). Energy levels and wave functions of Bloch electrons in rational and irrational magnetic fields. Phys. Rev. B.

[12] Albrecht C, Smet JH, von Klitzing K, Weiss D, Umansky V, Schweizer H (2001). Quantum Hall effect. Phys. Rev. Lett..

[13] Hatsugai Y, Kohmoto M (1990). Energy spectrum and quantum Hall effect on the square lattice with next-nearest-neighbor hopping. Phys. Rev. B.

[14] Han JH, Thouless DJ, Hiramoto H, Kohmoto M (1994). Critical and bicritical properties of Harper’s equation with next-nearest-neighbor coupling. Phys. Rev. B.

[15] Chang I, Ikezawa K, Kohmoto M (1997). Multifractal properties of the wave functions of the square-lattice tight-binding model with next-nearest-neighbor hopping in a magnetic field. Phys. Rev. B.

[16] Harper PG (1955). Single band motion of conduction electrons in a uniform magnetic field. Proc. Phys. Soc. A.

[17] Aubry S, André G (1980). Analyticity breaking and Anderson localization in incommensurate lattices. Ann. Isr. Phys. Soc..

[18] Lang L-J, Cai X, Chen S (2012). Edge states and topological phases in one-dimensional optical superlattices. Phys. Rev. Lett..

[19] Thouless DJ (1983). Bandwidths for a quasiperiodic tight-binding model. Phys. Rev. B.

[20] Ostlund S, Pandit R, Rand D, Schellnhuber HJ, Siggia ED (1983). One-dimensional Schrödinger equation with an almost periodic potential. Phys. Rev. Lett..

[21] Hiramoto H, Kohmoto M (1989). New localization in a quasiperiodic system. Phys. Rev. Lett..

[22] Khemani V, Sheng DN, Huse DA (2017). Two universality classes for the many-body localization transition. Phys. Rev. Lett..

[23] Lahini Y, Pugatch R, Pozzi F, Sorel M, Morandotti R, Davidson N, Silberberg Y (2009). Observation of a localization transition in quasiperiodic photonic lattices. Phys. Rev. Lett..

[24] Schreiber M (2015). Observation of many-body localization of interacting fermions in a quasi-random optical lattice. Science.

[25] Bordia P, Lüschen H, Schneider U, Knap M, Bloch I (2017). Periodically driving a many-body localized quantum system. Nat. Phys..

[26] Kraus YE, Lahini Y, Ringel Z, Verbin M, Zilberberg O (2012). Topological states and adiabatic pumping in quasicrystals. Phys. Rev. Lett..

[27] Grempel DR, Fishman S, Prange R (1982). Localization in an incommensurate potential: An exactly solvable model. Phys. Rev. Lett..

[28] Kohmoto M, Kadanoff LP, Tang C (1983). Localization problem in one dimension: Mapping and escape. Phys. Rev. Lett..

[29] Kohmoto M (1983). Metal-insulator transition and scaling for incommensurate systems. Phys. Rev. Lett..

[30] Levine D, Steinhardt PJ (1984). Quasicrystals: A new class of ordered structures. Phys. Rev. Lett..

[31] Thouless DJ (1988). Localization by a potential with slowly varying period. Phys. Rev. Lett..

[32] Hiramoto H, Kohmoto M (1989). Scaling analysis of quasiperiodic systems: Generalized harper model. Phys. Rev. B.

[33] Das Sarma S, He S, Xie XC (1988). Mobility edge in a model one-dimensional potential. Phys. Rev. Lett..

[34] Biddle J, Das Sarma S (2010). Predicted mobility edges in one-dimensional incommensurate optical lattices: An exactly solvable model of Anderson localization. Phys. Rev. Lett..

[35] Li X, Li X, Das Sarma S (2017). Mobility edges in one-dimensional bichromatic incommensurate potentials. Phys. Rev. B.

[36] Biddle J, Wang B, Priour DJ, Das Sarma S (2009). Localization in one-dimensional incommensurate lattices beyond the Aubry–André model. Phys. Rev. A.

[37] Aulbach C, Wobst A, Ingold G-L, Hänggi P, Varga I (2004). Phase-space visualization of metal-insulator transition. New J. Phys..

[38] Ganeshan S, Pixley JH, Sarma S. Das (2015). Nearest neighbor tight binding models with an exact mobility edge in one dimension. Phys. Rev. Lett..

[39] Liu F, Ghosh S, Chong YD (2015). Localization and adiabatic pumping in a generalized Aubry–André–Harper model. Phys. Rev. B.

[40] Li X, Ganeshan S, Pixley JH, Das Sarma S (2015). Many-body localization and quantum nonergodicity in a model with a single-particle mobility edge. Phys. Rev. Lett..

[41] Wang Y, Cheng C, Liu X-J, Yu D (2021). Many-body critical phase: Extended and nonthermal. Phys. Rev. Lett..

[42] Biddle J, Priour DJ, Wang B, Das Sarma S (2011). Localization in one-dimensional lattice with non-nearest-neighbor hopping. Phys. Rev. B.

[43] Martínez AJ, Porter MA, Kevrekidis PG (2018). Tight-binding model in optical waveguides. Philos. Trans. R. Soc. A.

[44] Domínguez-Castro GA, Paredes R (2019). The Aubry–André model as the hobbyhorse for understanding localization phenomenon. Eur. J. Phys..

[45] Guo C-X, Wang X, Hu H, Chen S (2023). Accumulation of scale-free localized states induced by local non-Hermiticity. Phys. Rev. B.

[46] Das KK, Christ J (2019). Realizing the Harper model with ultracold atoms in a ring lattice. Phys. Rev. A.

[47] Ni X, Chen K, Weiner M, Apigo DJ, Prodan C, Alù A, Prodan E, Khanikaev AB (2019). Observation of Hofstadter butterfly and topological edge states in reconfigurable quasi-periodic acoustic crystals. Commun. Phys..

[48] Gentile P, Cuoco M, Ortix C (2015). Edge states and topological insulating phases generated by curving a nanowire with Rashba spin-orbit coupling. Phys. Rev. Lett..

[49] Su WP, Schrieffer JR, Heeger AJ (1979). Solitons in polyacetylene. Phys. Rev. Lett..

[50] Su WP, Schrieffer JR, Heeger AJ (1980). Soliton excitations in polyacetylene. Phys. Rev. B.

[51] Meier EJ, An FA, Gadway B (2016). Observation of the topological soliton state in the Su–Schrieffer–Heeger model. Nat. Commun..

[52] Belopolski I, Xu S-Y, Koirala N, Liu C, Bian G, Strocov VN, Chang G, Neupane M, Alidoust N, Sanchez D, Zheng H (2017). A novel artificial condensed matter lattice and a new platform for one-dimensional topological phases. Sci. Adv..

[53] Chen X, Gu Z-C, Wen X-G (2011). Classification of gapped symmetric phases in one-dimensional spin systems. Phys. Rev. B.

[54] Chen X, Gu Z-C, Wen X-G (2011). Complete classification of one-dimensional gapped quantum phases in interacting spin systems. Phys. Rev. B.

[55] Zhu B, Lu R, Chen Sh (2014). Symmetry in the non-Hermitian Su–Schrieffer–Heeger model with complex boundary potentials. Phys. Rev. A.

[56] Li L, Chen Sh (2015). Topological properties of a generalized spin-orbit-coupled Su–Schrieffer–Heeger model. Europhys. Lett..

[57] Eliashvili M, Kereselidze D, Tsitsishvili G, Tsitsishvili M (2017). Edge states of a periodic chain with four-band energy spectrum. J. Phys. Soc. Jpn..

[58] Yan Z, Wan S (2014). Topological phases, topological flat bands, and topological excitations in a one-dimensional dimerized lattice with spin-orbit coupling. Europhys. Lett..

[59] Li L, Chen S (2015). Characterization of topological phase transitions via topological properties of transition points. Phys. Rev. B.

[60] Bahari M, Hosseini MV (2016). Zeeman-field-induced nontrivial topological phases in a one-dimensional spin-orbit-coupled dimerized lattice. Phys. Rev. B.

[61] Han YZ, Liu CS (2019). Topological phases of a non-Hermition coupled SSH ladder. Physica E.

[62] Li L, Xu Z, Chen S (2014). Topological phases of generalized Su–Schrieffer–Heeger models. Phys. Rev. B.

[63] Guo H, Chen Sh (2015). Quantum Monte Carlo study of hard-core bosons in Creutz ladder with zero flux. Phys. Rev. B.

[64] Maffei M, Dauphin A, Cardano F, Lewenstein M, Massignan P (2018). Topological characterization of chiral models through their long time dynamics. New J. Phys..

[65] Bahari M, Hosseini MV (2020). Topological properties of a generalized spin-orbit-coupled Su–Schrieffer–Heeger model. Physica E.

[66] Xie, D., Gou, W., Xiao, T., Gadway, B. & Yan, B. Topological characterizations of an extended Su–Schrieffer–Heeger model. *npj Quantum Inf.* **5**, 1 (2019).

[67] Bahari M, Hosseini MV (2019). One-dimensional topological metal. Phys. Rev. B.

[68] Li XP, Zhao E, Liu WV (2013). Topological states in a ladder-like optical lattice containing ultracold atoms in higher orbital bands. Nat. Commun..

[69] Cheon S, Kim TH, Lee SH, Yeom HW (2015). Chiral solitons in a coupled double Peierls chain. Science.

[70] Li C, Lin S, Zhang G, Song Z (2017). Topological nodal points in two coupled Su–Schrieffer–Heeger chains. Phys. Rev. B.

[71] Jangjan M, Hosseini MV (2021). Topological phase transition between a normal insulator and a topological metal state in a quasi-one-dimensional system. Sci. Rep..

[72] Jangjan M, Hosseini MV (2022). Topological properties of subsystem-symmetry-protected edge states in an extended quasi-one-dimensional dimerized lattice. Phys. Rev. B.

[73] Liu F, Wakabayashi K (2017). Novel topological phase with a zero berry curvature. Phys. Rev. Lett..

[74] Zhang S-L, Zhou Q (2017). Two-leg Su–Schrieffer–Heeger chain with glide reflection symmetry. Phys. Rev. A.

[75] Guo Z, Jiang J, Jiang H, Ren J, Chen H (2021). Observation of topological bound states in a double Su–Schrieffer–Heeger chain composed of split ring resonators. Phys. Rev. Res..

[76] Qian K, Apigo DJ, Padavić K, Ahn KH, Vishveshwara S, Prodan C (2023). Observation of Majorana-like bound states in metamaterial-based Kitaev chain analogs. Phys. Rev. Res..

[77] Ganeshan, S., Sun, K. & Sarma, S. Das. Topological zero-energy modes in gapless commensurate Aubry–André–Harper Models. *Phys. Rev. Lett.* **110**, 180403 (2013). 10.1103/PhysRevLett.110.180403 23683181

[78] Kraus YE, Zilberberg O (2012). Topological equivalence between the Fibonacci quasicrystal and the Harper model. Phys. Rev. Lett..

[79] Madsen KA, Bergholtz EJ, Brouwer PW (2013). Topological equivalence of crystal and quasicrystal band structures. Phys. Rev. B.

[80] Yahyavi M, Hetényi B, Tanatar B (2019). Generalized Aubry–André–Harper model with modulated hopping and p-wave pairing. Phys. Rev. B.

[81] Cestari, J. C. C. & Foerster, A. & Gusmão, M.A. Fate of topological states in incommensurate generalized Aubry–André models. *Phys. Rev. B* **93**, 205441 (2016).

[82] Padavić K, Hegde SS, DeGottardi W, Vishveshwara S (2018). Topological phases, edge modes, and the Hofstadter butterfly in coupled Su–Schrieffer–Heeger systems. Phys. Rev. B.

[83] Deng X, Ray S, Sinha S, Shlyapnikov GV, Santos L (2014). One-dimensional quasicrystals with power-law Hopping. Phys. Rev. Lett..

[84] Jangjan, M., Li, L., Foa Torres, L.E.F. & Hosseini, M.V. Topological phases of commensurate or incommensurate non-Hermitian Su–Schrieffer–Heeger lattices (submitted).

[85] Hughes TL, Prodan E, Bernevig BA (2011). Inversion-symmetric topological insulators. Phys. Rev. B.

[86] Jangjan M, Hosseini MV (2020). Floquet engineering of topological metal states and hybridization of edge states with bulk states in dimerized two-leg ladders. Sci. Rep..

[87] Kramer B, MacKinnon A (1993). Localization. Rep. Prog. Phys..

